# Real-life use of Rivaroxaban in the Netherlands: data from the Xarelto for Prevention of Stroke in Patients with Atrial Fibrillation (XANTUS) registry

**DOI:** 10.1007/s12471-017-1009-9

**Published:** 2017-07-03

**Authors:** R. Pisters, S. P. G. van Vugt, M. A. Brouwer, A. Elvan, W. L. ten Holt, P. A. G. Zwart, P. Kirchhof, H. J. G. M. Crijns, M. E. W. Hemels

**Affiliations:** 1 Department of Cardiology, Maastricht University Medical Centre, Maastricht, The Netherlands; 2 Department of Cardiology, Radboud University Medical Centre, Nijmegen, The Netherlands; 3 Department of Cardiology, Isala Hospital Zwolle, Zwolle, The Netherlands; 4 Department of Cardiology, Amstelland Hospital, Amstelveen, The Netherlands; 5 Department of Cardiology, Bernhoven Hospital, Uden, The Netherlands; 6 Centre for Cardiovascular Sciences, University of Birmingham, Birmingham, UK; 7 Department of Cardiology, Rijnstate Arnhem, Arnhem, The Netherlands

**Keywords:** Atrial fibrillation, Rivaroxaban, XANTUS, Non-VKA oral anticoagulation

## Abstract

**Background:**

The Xarelto for Prevention of Stroke in Patients with Atrial Fibrillation (XANTUS) registry investigated the safety and efficacy of the factor Xa inhibitor rivaroxaban. We studied the Dutch XANTUS cohort to a ssess drug safety and prescription patterns in the Netherlands.

**Methods:**

The XANTUS registry was designed as a European prospective, observational study among patients with non-valvular atrial fibrillation. Major bleeding and all-cause mortality were assessed every three months during a 1-year follow-up period. In this Dutch sub-cohort we were also specifically interested in dosing regimens and the incidence and reasons for temporary or permanent discontinuation.

**Results:**

Patients (*n* = 899) had a mean age of 69 (SD ± 9) years and 64.8% were male. The median CHA_2_DS_2_-VASc score was 2 (IQR 2–4) and the median HAS-BLED score was 2 (IQR 1–2). Major bleeding occurred in 19 patients (2.4 per 100 patient-years) and 8 patients (1.0 per 100 patient-years) died during the 1‑year follow-up period. According to renal function, label-discordant dosing was observed in 48 (8.3%) patients. Finally, 124 patients (13.8%) reported a temporary interruption of rivaroxaban treatment and 11.8% switched to another oral anticoagulant therapy after permanent discontinuation of rivaroxaban.

**Conclusion:**

In the Dutch subset of the XANTUS registry, we observed low rates of major bleeding and label-discordant dosing and high persistence rates during one year of follow-up in patients receiving rivaroxaban in routine clinical practice. However, documenting the motivation of novel oral anticoagulant (NOAC) type and dose is essential to study label-discordant prescription, a potential safety paradox and identify patient characteristics to optimise NOAC use and adherence.

## Introduction

The landscape concerning antithrombotic treatment of patients with non-valvular atrial fibrillation (AF) has changed in the last decade. First, anticoagulant treatment showed its superiority as compared with antiplatelet agents for stroke prevention [1–3]. Second, new oral anticoagulant agents have been approved as an alternative for vitamin K antagonists (VKAs) [4–7]. As a class, these agents, commonly referred to as non-VKA oral anticoagulants (NOACs), reduce the risk of intracranial haemorrhage by approximately 50% and of major bleeding by approximately 15% in comparison with VKA treatment [8]. Consequently, the recently updated European guidelines on the management of AF recommend the use of NOAC treatment over VKA treatment in eligible patients [9].

In the context of a long history of well-monitored VKA treatment, real-world data on compliance and management of complications were topics of interest at the time of the implementation of the NOACs. Therefore, at the request of the Dutch Ministry of Health, Welfare and Sports, a guidance document was published in November 2012 to facilitate a gradual implementation of the NOACs in daily clinical practice [10]. In accordance with other NOAC registries [11–15], in the Xarelto for Prevention of Stroke in Patients with Atrial Fibrillation (XANTUS) registry the investigators collected data on the safety of rivaroxaban in a ‘real-world’ setting. In addition, ischaemic complications, drug dosing and discontinuation rates were assessed [16]. In the present study, we evaluated the abovementioned outcome measures for the Dutch population of the XANTUS registry.

## Methods

XANTUS is a European prospective, post-authorisation, observational phase IV study in patients with non-valvular AF treated with rivaroxaban for stroke prevention. Its design has been published previously [17].

### Study population and follow-up

In brief, patients were eligible if they were diagnosed with non-valvular AF, started rivaroxaban therapy and provided written informed consent. All patients were screened sequentially and data were documented in an anonymous log file. Enrolment in the XANTUS registry took place between June 2012 and December 2013. For the purpose of this study we selected patients enrolled in the Netherlands. Decisions with regard to rivaroxaban prescription (e. g. dose selection, interruption and discontinuation) were at the discretion of the treating physician.

### Study outcomes

The primary study outcomes were related to the safety of rivaroxaban and comprised major bleeding events and all-cause mortality [16]. Major bleeding was defined according to the criteria suggested by the International Society of Thrombosis and Haemostasis (ISTH) [18]. A bleeding event was considered fatal when death occurred within 30 days of the bleeding event.

Secondary outcomes included clinically overt thromboembolic events (stroke, non-central nervous systemic embolism, transient ischaemic attack, myocardial infarction) and any non-major bleeding.

With regard to guideline adherence, we evaluated the dosing regimen in the perspective of renal function. Furthermore, we evaluated temporary and permanent discontinuation rates, including the reported reasons.

### Statistical considerations

Given the exploratory purpose of the study, statistical analyses were descriptive. Data were depicted as means and standard deviations (SD) or medians and interquartile ranges (IQR), whichever was appropriate. Categorical variables were depicted as frequencies and percentages.

## Results

### Baseline characteristics

Among the 6784 participants of the XANTUS registry, a total of 899 (13.3%) were enrolled in the Netherlands. The mean age of the Dutch patients was 69.2 (SD ± 8.9) years and 583 (64.8%) were male (Table 1). With regard to risk scores, the median CHA_2_DS_2_-VASc score was 2 (IQR 2–4) and the median HAS-BLED score was 2 (IQR 1–2). Data concerning renal function were available in 580 (64.5%) patients. A total of 53 (5.9%) patients had a creatinine clearance of <50 ml/min.

**Table 1:** Baseline characteristics of Dutch patients in the XANTUS study (*n* = 899)

Age (years), mean ± SD		69.2 ± 8.9
	≥75, *n* (%)	254 (28.3%)
Gender (male), *n* (%)		583 (64.8%)
Weight (kg), mean ± SD		85.8 ± 17.1
Creatinine clearance (ml/min), *n* (%)		
	15–29	5 (0.6%)
	30–49	48 (5.3%)
	≥50	527 (58.6%)
	Missing	319 (35.5%)
Atrial fibrillation type, *n* (%)		
	First diagnosed	170 (18.9%)
	Paroxysmal	473 (52.6%)
	Persistent	98 (10.9%)
	Permanent	158 (17.6%)
CHADS_2_ score, median (IQR)		1 (1–2)
	<2	556 (61.8%)
	≥2	343 (38.2%)
CHA_2_DS_2_-VASc score, median (IQR)		2 (2–4)
HAS-BLED score, median (IQR)		2 (1–2)
	≥3	161 (17.9%)
Prior stroke/TIA/non-CNS SE, *n* (%)		107 (11.9%)
Congestive heart failure, *n* (%)		58 (6.5%)
Hypertension, *n* (%)		550 (61.2%)
Coronary artery disease, *n* (%)		93 (10.3%)
Peripheral artery disease, *n* (%)		47 (5.2%)
Diabetes mellitus, *n* (%)		143 (15.9%)
Prior use of antithrombotic therapy, *n* (%)		779 (86.7%)
	VKA	559 (62.2%)
	Direct thrombin inhibitor	14 (1.6%)
	ASA	120 (13.3%)

*SD* standard deviation, *BMI* body mass index, *IQR* interquartile range, *TIA* transient ischaemic attack, *CNS* central nervous system, *SE* systemic embolism, *VKA* vitamin K antagonist, *ASA* acetylsalicylic acid

Creatinine clearance calculated using the Cockcroft-Gault formula

Before enrolment in the registry, 779 patients (86.7%) were using antithrombotic therapy, which included VKA in 576 (64.1%) patients. Acetylsalicylic acid as monotherapy was reported in 120 (13.3%) patients prior to treatment with rivaroxaban (Table 1).

With regard to follow-up, 764 (85.0%) fulfilled the 1‑year observation period.

### Study outcomes

A total of 21 major bleeding events were observed in 19 (2.1%) patients (Table 2). Bleeding events occurred most frequently in the gastrointestinal tract, followed by intracranial bleeding (Table 3). Gastrointestinal bleeding was reported 9 times in 7 patients. Fifteen patients reported unscheduled contact with a physician, in 10 patients medical intervention was necessary. With regard to all-cause mortality, a total of 8 (0.9%) patients died during follow-up. Three deaths were considered to be caused by fatal bleeding and 2 were cardiovascular in origin. In addition, cancer, infectious disease and an unclassified cause were reported once.

**Table 2:** Study endpoints

		Incidence proportion, *n* (%)	Incidence rate, events per 100 patient-years (95% CI)
*Primary outcomes*	Major bleeding	19 (2.1%)	2.4 (1.4–3.7)
	All-cause mortality	8 (0.9%)	1.0 (0.4–2.0)
*Secondary outcomes*	Thromboembolic event	13 (1.4%)	1.6 (0.9–2.8)
	Non-major bleeding	142 (15.8%)	19.6 (16.5–23.1)

*CI* confidence interval

**Table 3:** Bleeding complications according to location

		Number of patients (%)
*Major bleeding*		19 (2.1%)
	Gastrointestinal	7 (0.8%)
	Intracranial	4 (0.4%)
	Genitourinary	2 (0.2%)
	Conjunctival	1 (0.1%)
	Intraocular	1 (0.1%)
	Musculoskeletal	1 (0.1%)
	Skin	1 (0.1%)
	Surgery site	1 (0.1%)
	Not reported	1 (0.1%)
*Non-major bleeding*		142 (15.8%)
	Nasal	56 (6.2%)
	Genitourinary	27 (3.0%)
	Skin	26 (2.9%)
	Gastrointestinal	21 (2.3%)
	Other	12 (1.3%)

Concerning the secondary outcomes, 15 thromboembolic events were reported in 13 (1.4%) patients. These comprised 4 ischaemic strokes, 4 myocardial infarctions and 7 transient ischaemic attacks.

Any non-major bleeding occurred 195 times in 142 (15.8%) patients (Table 3). A total of 68 patients reported unscheduled contact with a physician; subsequent medical intervention was taken in 26 patients. With regard to the impact of bleeding, patient discomfort (i. e. pain or impairment of daily life activities) was reported in 14 (73.7%) cases with major bleeding events and in 38 (26.8%) in patients with non-major bleeding complications.

### Rivaroxaban prescription and dosing regimens

Rivaroxaban was prescribed in a dose of 20 milligrams (mg) once daily in 817 (90.8%) of the patients. These patients had a median HAS-BLED score of 2 (1–2) and 133 (16.3%) had a HAS-BLED score ≥3. Among the patients with a daily dose of 15 mg, the median HAS-BLED score was 2 (2–3) and 26 (41.8%) had a HAS-BLED score ≥3. The remaining 3 patients received an initial daily dose of 10 mg.

**Fig. 1:**
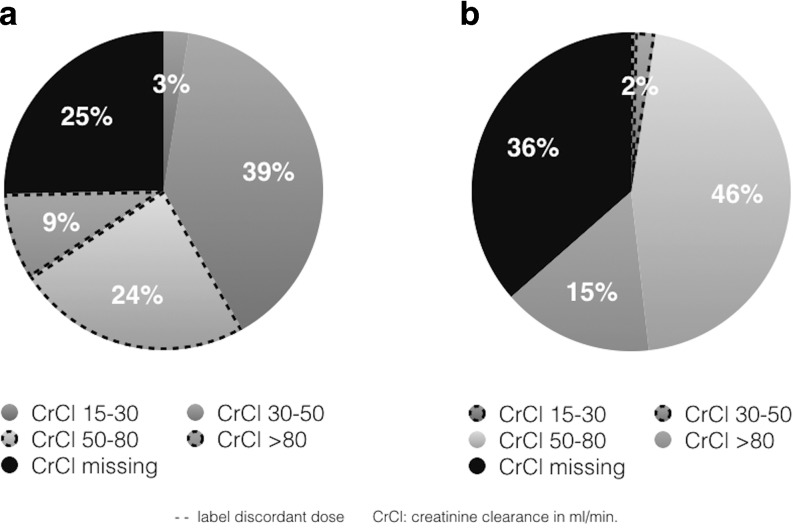
Renal function according to initially prescribed dose *CrCl* creatinine clearance

According to renal function, label-discordant dosing was observed in 48 (8.3%) patients; 2 patients received a daily dose of 10 mg, 26 patients received 15 mg discordantly and 20 patients received 20 mg not according to label (Fig. 1). Major bleeding occurred in 17 (2.1%) and 2 (2.5%) patients treated with rivaroxaban 20 mg and 15 mg, respectively.

### Discontinuation rates

During follow-up, 124 patients (13.8%) reported a temporary interruption of rivaroxaban treatment. Reasons included surgery (*n* = 60), bleeding (*n* = 35) and non-bleeding adverse events (*n* = 29). The median duration of interruption was 3 days (IQR 2–8).

**Fig. 2:**
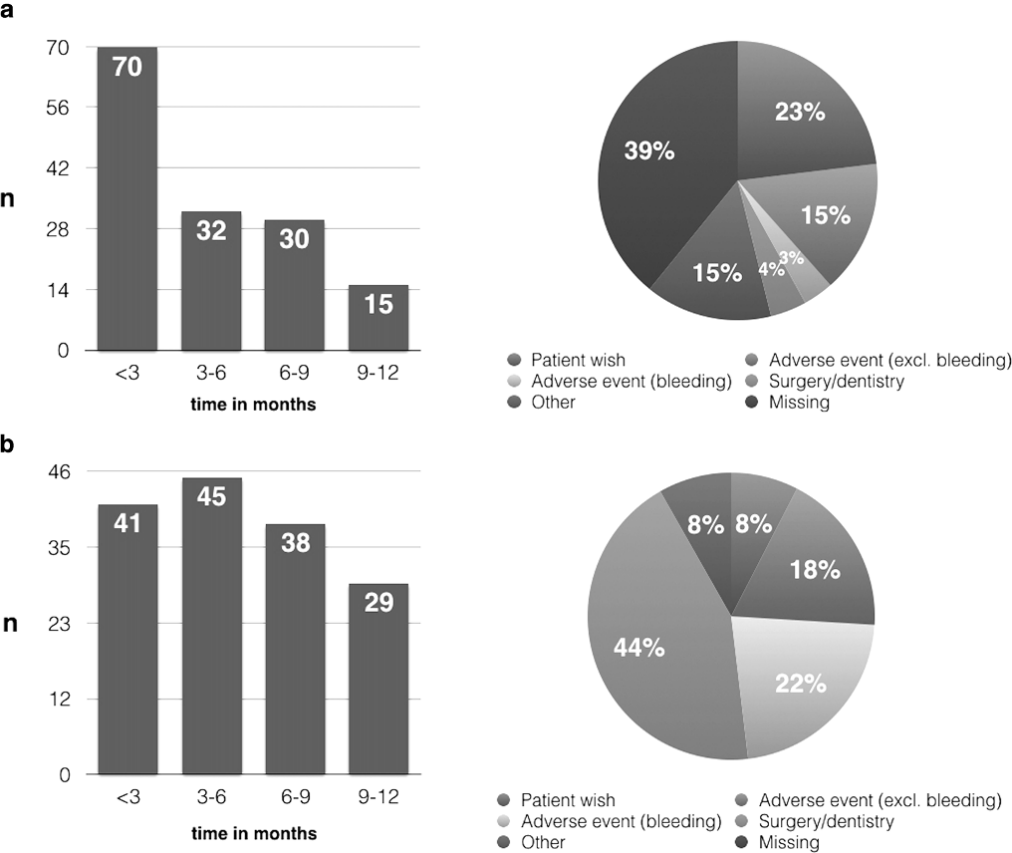
Permanent (**a**) and temporary (**b**) reason of discontinuation among patients who stopped study treatment

Study treatment was discontinued in 147 (16.4%) patients, half of whom discontinued within 3 months after initiation (Fig. 2). The main documented reasons for permanent discontinuation were patient decision (*n* = 33, 23%) or an adverse event (*n* = 27, 19%) (Fig. 2a). The majority of patients (*n* = 84) who stopped study treatment switched to VKAs, followed by a switch to a different NOAC (*n* = 22).

## Discussion

### Main outcomes and patient population

In this substudy of the XANTUS registry we assessed the safety of rivaroxaban in a ‘real-world’ situation in the Netherlands. The Dutch subgroup consisted of less permanent AF patients compared with the overall XANTUS cohort [16]. Subsequently, this largely explains the observed differences in patient characteristics between the two cohorts, i. e. a younger and healthier Dutch subgroup. The inclusion of more paroxysmal AF patients in the Netherlands likely relates to national policy which advocates the initial use of NOACs in first detected AF opposed as to actively switching patients from VKAs to NOACs. Among the 899 patients major bleeding rates, which have been published previously, were low and comparable with the overall XANTUS cohort. Similar observations were made for the rates of thromboembolic events and all-cause mortality [16]. Furthermore, we observed that over 90% of patients were treated according to label in daily clinical practice, and that 1‑year persistence rate was over 80%.

Major bleeding events were observed less frequently than in the overall XANTUS cohort (2.1/100 patient-years) and the ROCKET-AF study (3.5/100 patient-years). In both the overall XANTUS cohort and the Dutch subset over 40% of the major bleeding complications had a gastrointestinal origin. Among the patients randomised to rivaroxaban treatment in the ROCKET-AF trial, gastrointestinal bleeding comprised over 50% (224/395) of the major bleeding complications. The event rate for intracranial haemorrhage, often considered to be the most devastating bleeding complication, accounted for 20% of the major bleeding complications in both the overall and the Dutch XANTUS cohort, as compared with 15% in the ROCKET-AF trial population.

With regard to ischaemic complications and mortality, event rates in the Dutch XANTUS cohort were low compared with the ROCKET-AF population. The differences in event rates for ischaemic stroke (0.5 vs 1.4/100 patient-years) and all-cause mortality (1.0 vs 1.9/100 patient-years) might be explained by important differences in baseline characteristics. Of the Dutch XANTUS patients, 28% were aged 75 years or over, which is less than in the overall XANTUS cohort (38%) or ROCKET-AF population (43%). Among the ROCKET-AF population, all patients had a CHADS_2_ score of at least 2 points. In contrast, only 38% of the Dutch XANTUS patients had a CHADS_2_ score ≥2. As for renal function, 6% had a creatinine clearance below 50 ml/min, whereas these percentages were 9% and 20% for the overall XANTUS and ROCKET-AF cohorts, respectively. Note that the ROCKET-AF trial intended to include a high-risk population. As such, the inclusion criteria were different from the XANTUS registry. Initial prescription of NOACs in a low-risk population is in line with the recommendations of the Dutch Ministry of Health, Welfare and Sport, proposing a careful introduction of NOACs in the Netherlands. Noteworthy, most treatment switches from rivaroxaban were to VKAs (84/143), indicating the relative novelty of NOACs during the study period. In addition, the gradual uptake of NOAC treatment in the Netherlands has been described recently [19].

### Dosing issues

In 8% of the Dutch XANTUS participants, rivaroxaban was prescribed in an inappropriate dose. As stated in the summary of product characteristics (SmPC), dose recommendations are particularly based upon renal function. However, prescribing physicians were probably influenced by other factors. First, previous guidelines recommended the lower NOAC dose for patients with a HAS-BLED score ≥3 [20]. Current guidelines do not recommend dose reductions based on bleeding risk scores [9].

It is important to state that the rationale behind the dose-reduction strategies in the randomised trials was to avoid overdosing in specific patient groups. In a recently published sub-analysis of the ARISTOTLE trial, the authors state that inappropriate dosing could lead to preventable ischaemic strokes and that patients therefore should be treated with the studied dose [21]. A similar statement is found in the European Society of Cardiology guidelines on AF management [9]. Consequently, other perceived bleeding risk factors, including a prior bleeding event or concomitant use of antiplatelet agents, should not per se lead to prescription of a reduced dose. With regard to prior bleeding, exploratory data from the RE-LY trial did not support a dose-lowering strategy after a bleeding episode [22].

Nevertheless data on creatinine clearance were missing in over one-third of the patients. Whether this is because of clinical judgment (no need to measure the creatinine clearance) or due to the observational nature of the study (not reported) remains speculative. The observed low rates of stroke and bleeding and limited use of low-dose rivaroxaban support the former. Dose reduction during follow-up was uncommon, perhaps this is due to limited follow-up of creatinine clearance measurements or to the relatively low risk profile of the population.

All in all, these data advocate to prescribe NOACs in doses as evaluated in trial settings and be aware of the safety paradox: a lower (N)OAC dose does not equal a low(er) risk of bleeding and preserved efficacy [22, 23].

### Discontinuation

Despite good treatment satisfaction, 1 out of 6 patients permanently discontinued treatment with rivaroxaban during the follow-up period, with a peak during the first quarter. Although the motivation for discontinuation of rivaroxaban was missing in a substantial number of patients, patient decisions and adverse events were reported. Nonetheless, a persistence rate over 80% was reported in the Dresden registry as well, in which bleeding complications and non-bleeding side effects accounted for the majority of treatment discontinuations [24].

The majority of patients (106/147) switched to either VKA treatment (*n* = 84) or another NOAC (*n* = 22) after rivaroxaban discontinuation. The relatively high number of patients switching to VKA treatment should be placed in a time perspective. The remaining patients might have had a temporary indication for anticoagulation therapy. Given the considerable proportion of paroxysmal AF and young (e. g. <65 years of age) patients together with a CHA_2_DS_2_-VASc score of 0 or 1, this temporary indication could be related to a rhythm control strategy (e. g. peri-ablation or cardioversion). Despite the relatively high percentage (13%) of patients treated with antiplatelet therapy for stroke prevention before study entry, observations from this study were reassuring, as a switch from rivaroxaban to antiplatelet therapy was uncommon (<1%). Treatment was interrupted temporarily in 13.8% of the patients in the study cohort, a proportion slightly higher compared with the overall XANTUS cohort (8.8%). As compared with the ROCKET-AF population (2165/7131 patients treated with rivaroxaban) interruption rates were similar, as were the main reasons for interruption, which included surgery and bleeding events [25].

## Limitations

Although the first real-world data concerning NOAC use in the Netherlands are very valuable, they should be interpreted in the context of guideline recommendations at the time the study was conducted (2012–2013). At present, NOACs are prescribed more liberally, and the current ESC guideline has endorsed NOACs as the preferred treatment [9]. Second, we should acknowledge that data on renal function were not available at the time of prescribing rivaroxaban in one-third of the patients. Again, this observation deserves particular clinical attention as dose reduction in the ROCKET-AF randomised controlled trial (RCT) and dose recommendations in the SmPC rely particularly on renal function. Finally, the study cohort was a relatively healthy population. Initial selection of patients even more healthy than in the respective RCT is common with the introduction of new drugs. Although this exemplifies real-life, we must be aware of underrepresentation of subgroups of patients in an RCT and ‘real-life’ setting. For instance, whether the observations can be extrapolated to a very high-risk population is questionable. This question will probably be answered by the randomised FRAIL-AF trial, which will be conducted in the Netherlands [26].

## Conclusion

In the Dutch subset of the XANTUS registry, we observed low rates of major bleeding and label-discordant dosing and high persistence rates during one year of follow-up in patients receiving rivaroxaban in routine clinical practice. However, documenting the motivation of NOAC type and dose is essential to study label-discordant prescription, a potential safety paradox and identify patient characteristics to optimise NOAC use and adherence.
